# Vitamin D Modulates Humoral Responses to SARS-CoV-2 Vaccination in Autoimmune Thyroiditis: An Endocrine–Immune Perspective Supported by Network Pharmacology, Molecular Docking, and Molecular Dynamics Simulations

**DOI:** 10.3390/ijms27052208

**Published:** 2026-02-26

**Authors:** Nawel Zerouak, Salma Hentabli, Abderrahmane Zitouni, Mouna Lehassani, Hamza Hentabli, Mohamed Anis Haroun, Mammar Khames, Karine Benachour, Yassine Amrani, Mustapha Oumouna

**Affiliations:** 1 Laboratory of Biology and Experimental Pharmacology, Faculty of Sciences, Dr. Yahia Fares University, Medea 26000, Algeria; zerouak.nawel@univ-medea.dz (N.Z.); hentabli.selma@gmail.com (S.H.); benachourkarine1@gmail.com (K.B.); 2 Department of Biology and Ecology, Faculty of Sciences, Dr. Yahia Fares University, Medea 26000, Algeria; 3 Computational Chemistry and Biophysics Group Drug Design, Department of Chemistry, University of Missouri-Columbia, Columbia, MI 65211, USA; hhhx5@missouri.edu; 4 Department of Respiratory Sciences, College of Life Sciences and NIHR Biomedical Research Centre (Respiratory Theme), Glenfield Hospital, Leicester LE1 7RH, UK

**Keywords:** vitamin D, autoimmune thyroiditis, COVID-19 vaccination, humoral immune responses, network pharmacology, endocrine–immune crosstalk, autoimmune endocrinology, molecular docking, molecular dynamics

## Abstract

Autoimmune thyroiditis (AIT) is characterized by dysregulated endocrine–immune interactions, and vitamin D has been proposed as a potential immunomodulatory factor influencing vaccine-induced immune responses. This study investigated the association between serum vitamin D status and humoral responses to SARS-CoV-2 vaccination in patients with AIT, while exploring potential molecular mechanisms using network pharmacology, molecular docking and Molecular Dynamics (MD) simulations. Patients were stratified according to serum 25-hydroxyvitamin D levels as deficient, insufficient, or sufficient. Anti–spike receptor-binding domain (RBD) IgG titers, thyroid autoantibodies, and thyroid-stimulating hormone levels were measured. In parallel, vitamin D_3_ related molecular targets were integrated with AIT-associated genes, followed by protein–protein interaction analysis, molecular docking and MD simulations were performed to assess the interactions between vitamin D_3_ (cholecalciferol) and selected key proteins. An inverse correlation was observed between serum vitamin D levels and anti-RBD IgG titers (*p* = 0.0013), with higher antibody responses detected in vitamin D-deficient patients. Network pharmacology analysis highlighted CYP19A1, CYP17A1, and ESR1 as prioritized targets associated with steroid hormone biosynthesis and endocrine signaling pathways. Molecular docking showed compatible binding of vitamin D_3_ to these proteins, while MD simulations supported the structural stability of the complexes over time. Collectively, these findings suggest that vitamin D status may influence post-vaccination humoral immune responses in AIT, potentially through modulation of endocrine–immune crosstalk. Further longitudinal and mechanistic studies are required to clarify causality and clinical relevance.

## 1. Introduction

Autoimmune thyroiditis (AIT), most commonly represented by Hashimoto’s thyroiditis, is a chronic endocrine autoimmune disorder characterized by progressive immune-mediated destruction of the thyroid gland [1]. The disease is typically associated with elevated concentrations of anti-thyroid peroxidase (anti-TPO) and anti-thyroglobulin (anti-TG) antibodies in the serum and predominantly affects women. Its pathogenesis reflects a multifactorial interplay between genetic susceptibility, environmental influences, hormonal factors, and dysregulated immune responses [2].

The COVID-19 pandemic has highlighted the vulnerability of individuals with underlying immune-mediated disorders to altered immune responses following both infection and vaccination. SARS-CoV-2 infection has been associated with thyroid dysfunction, including subacute thyroiditis, hypothyroidism, and exacerbation of pre-existing autoimmune thyroid disease [3]. These effects have been attributed, at least in part, to viral entry mechanisms involving ACE2 and TMPRSS2, which are expressed in thyroid tissue, as well as to post-infectious immune dysregulation [4].

Vitamin D exists in two principal forms, D_2_ (ergocalciferol) and D_3_ (cholecalciferol), and undergoes sequential hydroxylation in the liver (via CYP2R1) and the kidney (via CYP27B1) to form the biologically active metabolite (1,25-dihydroxyvitamin D_3_ [1,25(OH)_2_D_3_]) [5,6]. Beyond its classical role in calcium and bone metabolism, vitamin D exerts important immunomodulatory effects through genomic and non-genomic effects mediated by the vitamin D receptor (VDR). The VDR is widely expressed in immune cells, including T and B lymphocytes, macrophages, and dendritic cells, underscoring vitamin D’s role in regulating both innate and adaptive immune responses [7].

**Figure 1:**
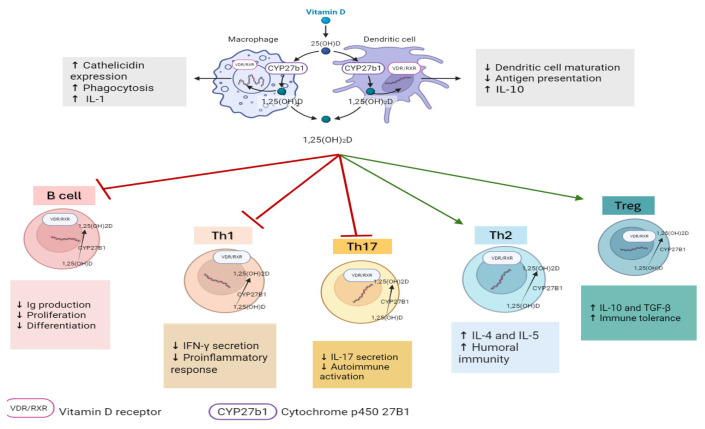
Immunomodulatory effects of vitamin D on innate and adaptive immune responses. 1,25(OH)_2_D: 1,25-dihyroxyvitamin D; 25(OH)D: 25-hyroxyvitamin D; INF-γ: interferon-γ; IL: interleukin; Th1: T helper 1; Th2: T helper 2; Th17: T helper17; Treg: regulatory T cell; TNF- α: Tumor necrosis factor- α; TGF-β: Transforming Growth Factor Beta.

Vitamin D influences immune homeostasis by modulating cytokine production, antigen presentation, and lymphocyte differentiation. It has been shown to suppress pro-inflammatory mediators such as IL-6, TNF-α, and IL-17 while promoting anti-inflammatory cytokines, including IL-10 and TGF-β, and favors regulatory T cell (Treg) differentiation over Th1 and Th17 polarization. In addition, vitamin D affects B-cell activation, plasma cell differentiation, and antibody production [8,9]. These immunoregulatory actions are particularly relevant in autoimmune thyroiditis, where vitamin D deficiency has been associated with increased disease severity and higher thyroid autoantibody titers. The principal immunological pathways influenced by vitamin D are summarized in Figure 1.

SARS-CoV-2 infection and vaccination are known to induce robust immune activation, which in some individuals may unmask or exacerbate autoimmune phenomena [10,11]. Proposed mechanisms include molecular mimicry between viral and host antigens [12], bystander activation of autoreactive T cells [13], and disruption of regulatory immune networks [14], and prolonged post-viral inflammation affecting the hypothalamic–pituitary–thyroid axis. In patients with autoimmune thyroiditis, such immune perturbations may influence vaccine-induced responses, including humoral immunity [15].

Recent studies suggest that vitamin D status may be associated with variability in vaccine-induced antibody responses [16]. However, findings across populations and disease contexts remain heterogeneous. In autoimmune conditions, exaggerated antibody responses may reflect underlying immune dysregulation rather than enhanced protective immunity [17,18,19]. The biological mechanisms linking vitamin D status, endocrine regulation, and post-vaccination humoral responses in autoimmune thyroiditis are not yet fully understood.

In addition to its direct immunomodulatory effects, vitamin D is closely interconnected with endocrine pathways involved in steroid hormone biosynthesis and hormone receptor signaling. These endocrine pathways are known to influence immune function, sex-specific differences in autoimmunity, and B-cell activity. Autoimmune thyroiditis displays a strong sex-hormone bias and is influenced by estrogen, androgen, and metabolic pathways. Therefore, exploring the molecular crosstalk between vitamin D signaling and endocrine–immune regulation is essential for understanding vaccine-related immune responses in AIT. Network pharmacology provides a systems-level approach to explore such complex interactions by integrating disease-associated genes, molecular targets, and signaling pathways. When combined with molecular docking, this strategy enables hypothesis-generating exploration of potential molecular interactions that may link vitamin D status with endocrine modulation and humoral immune responses.

The objective of this study was to investigate the association between serum vitamin D concentrations and anti-SARS-CoV-2 receptor-binding domain (RBD) IgG antibody levels in patients with autoimmune thyroiditis following COVID-19 vaccination. To complement the clinical findings, network pharmacology and molecular docking analyses were employed to explore potential endocrine-related molecular pathways through which vitamin D may be linked to immune regulation in autoimmune thyroiditis. By integrating clinical observations with in silico analyses, this work aims to provide a broader perspective on endocrine–immune crosstalk in the context of vaccination in autoimmune disease.

## 2. Results

### 2.1. Clinical and Serological Characteristics

**Figure 2:**
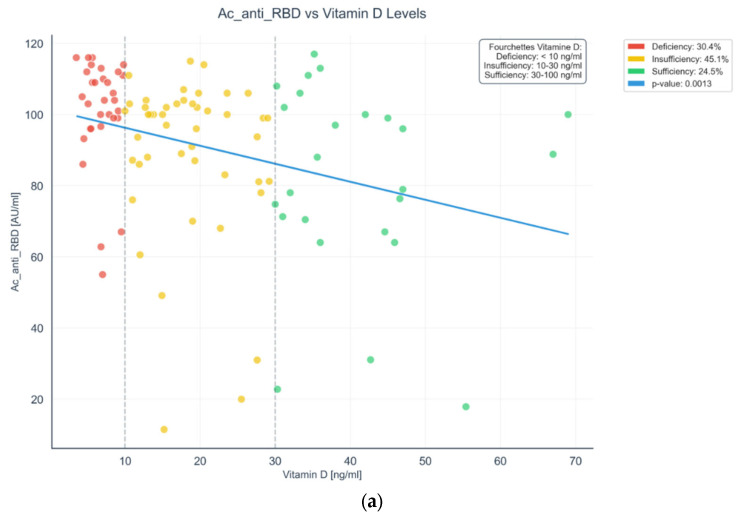

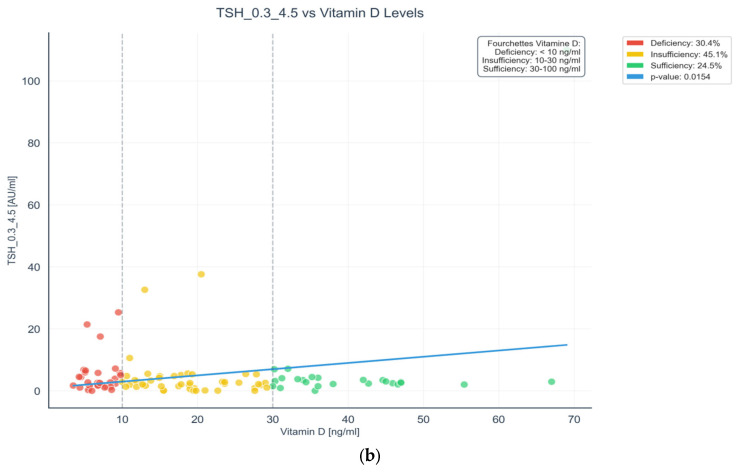
(**a**) Scatter plot of serum 25(OH)D levels versus anti-SARS-CoV-2 S-RBD IgG titers in autoimmune thyroiditis patients post-vaccination. (**b**) Scatter plots and distribution of 25(OH) vitamin D levels, TSH titers in autoimmune thyroiditis patients after COVID-19 vaccination. Abbreviations: 25(OH)D, 25-hydroxyvitamin D; SARS-CoV-2, Severe Acute Respiratory Syndrome Coronavirus 2; S-RBD, Spike Receptor-Binding Domain; IgG, Immunoglobulin G; TSH, Thyroid-Stimulating Hormone; AIT, Autoimmune Thyroiditis.

To evaluate the interplay between vitamin D status, immune response, and thyroid function in patients with autoimmune thyroiditis following COVID-19 vaccination, we examined key clinical and immunological markers. Figure 2a presents a scatter plot illustrating the correlation between serum 25(OH) D levels and anti-SARS-CoV-2 S-RBD IgG antibody titers, reflecting the humoral immune response. The association between serum 25(OH)D levels and anti-RBD IgG titers was statistically assessed using Spearman’s rank correlation analysis. In contrast, Figure 2b displays the distribution of 25(OH) D concentrations alongside TSH levels, allowing a comparative overview of vitamin D status and thyroid function across the study population.

This plot displays the relationship between serum 25-hydroxyvitamin D [25(OH)D] concentrations (ng/mL) and anti-SARS-CoV-2 spike receptor-binding domain (S-RBD) IgG antibody levels (AU/mL) in patients with autoimmune thyroiditis following COVID-19 vaccination. Patients are color-coded by vitamin D status: deficient (<10 ng/mL, red; 30.4%), insufficient (10–30 ng/mL, yellow; 45.1%), and sufficient (≥30 ng/mL, green; 24.5%). A significant inverse correlation was observed (*p* = 0.0013), with higher antibody titers in vitamin D-deficient individuals and lower, more stable responses in those with sufficient levels. The blue trend line represents the negative correlation, suggesting that vitamin D status may influence the magnitude and regulation of vaccine-induced humoral responses in this population.

Scatter plot showing serum 25(OH)D levels (ng/mL) against TSH levels (µIU/mL) in patients with autoimmune thyroiditis following COVID-19 vaccination. The X-axis represents vitamin D concentration categories, and the Y-axis shows TSH values (normal range: 0.3–4.5 µIU/mL). A significant positive correlation was observed (*p* = 0.0154). Higher TSH levels were observed in patients with vitamin D deficiency (<10 ng/mL), whereas patients with sufficient vitamin D (≥30 ng/mL) had more stable TSH levels.

### 2.2. Distribution of Vitamin D and Anti-RBD IgG and Thyroid Autoimmunity Markers by Age

**Figure 3:**
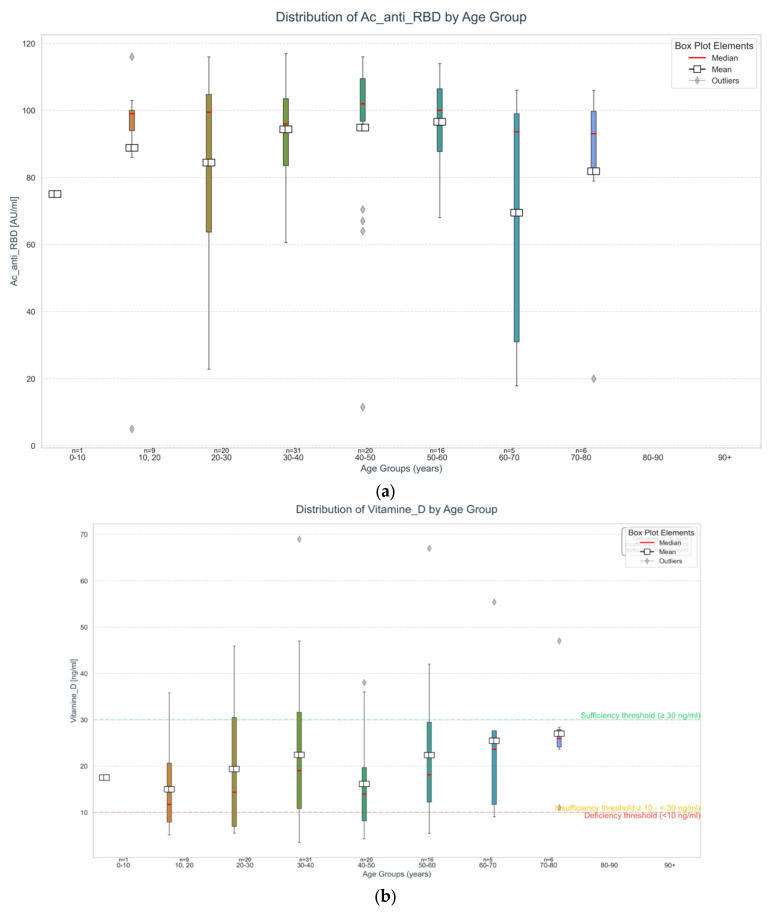

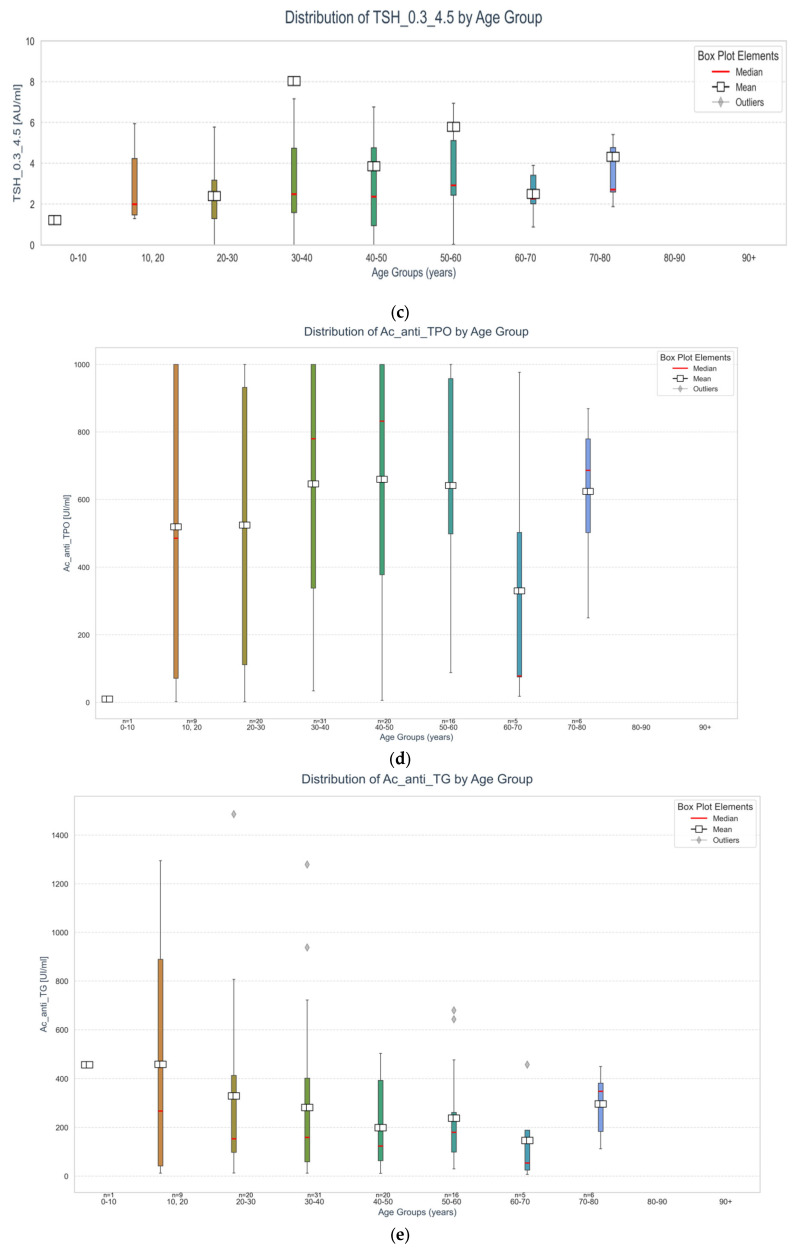
(**a**) Distribution of anti-RBD IgG antibody levels across age groups in autoimmune thyroiditis patients post-vaccination. (**b**) Distribution of 25(OH) D across different age groups in autoimmune thyroiditis patients. (**c**) Distribution of TSH across different age groups in autoimmune thyroiditis patients. (**d**) Distribution of AC anti TPO across different age groups in autoimmune thyroiditis patients. (**e**) Distribution of AC anti TG across different age groups in autoimmune thyroiditis patients.

To examine the influence of age on immunological and endocrine profiles in patients with autoimmune thyroiditis post-COVID-19 vaccination, we assessed the distribution of several biomarkers across different age groups. As shown in Figure 3a, anti-RBD IgG antibody levels demonstrated variability with age, suggesting possible age-related differences in humoral response. In Figure 3b, serum 25(OH)D concentrations are presented across the same age groups, revealing distinct patterns in vitamin D status. Figure 3c illustrates the distribution of TSH levels, while Figure 3d,e depict the levels of anti-TPO and anti-TG antibodies, respectively. Statistical comparisons across these age-defined groups were performed using the Kruskal–Wallis test to identify significant differences in biomarker distributions. Together, these age-stratified analyses provide insight into how immune activation, endocrine function, and autoantibody production may vary across the lifespan in this patient population.

Box plot illustrating the distribution of anti-SARS-CoV-2 spike receptor-binding domain (S-RBD) IgG antibody concentrations (AU/mL) stratified by age group in patients with autoimmune thyroiditis following COVID-19 vaccination. Younger individuals (10–60 years) exhibited higher and more consistent antibody responses, with median values nearing 100 AU/mL. In contrast, older patients (≥60 years), particularly those in the 60–70 age group, showed greater variability and a wider interquartile range. The presence of multiple outliers across all the groups highlights individual variability in humoral immune responses. These findings suggest a potential age-related decline in vaccine-induced immunity in this population.

Box plot showing the distribution of serum 25(OH)D levels across different age groups in patients with autoimmune thyroiditis. Deficiency (<10 ng/mL) and sufficiency (≥30 ng/mL) thresholds are indicated. Most age groups had median vitamin D levels below the sufficiency cutoff, reflecting widespread insufficiency. Greater variability was observed in individuals aged 10–60 years, with some reaching sufficient levels, whereas older adults (≥60 years) showed lower and more stable vitamin D concentrations. Outliers above 50 ng/mL may reflect individual differences in supplementation or sun exposure. These results highlight the need for monitoring vitamin D status, particularly among older adults.

The box plot illustrates TSH levels across different age groups in patients with autoimmune thyroiditis. While overall values remain within the normal range (0.3–4.5 µIU/mL), a slight increase is observed in the 50–60 and 70–80 age groups. Greater variability is noted among younger individuals (10–50 years), whereas older age groups show more consistent values. The presence of outliers suggests occasional cases of thyroid dysregulation, potentially influenced by age-related hormonal, metabolic, or autoimmune factors.

The box plot illustrates the distribution of anti-thyroid peroxidase (anti-TPO) antibody levels across different age groups. Individuals aged 10–60 years exhibit marked variability, with a wide interquartile range (IQR) and numerous outliers, reflecting heterogeneous autoimmune activity. In contrast, a narrower IQR is observed beyond 60 years of age, suggesting a possible decline in autoimmune reactivity with aging. Median values remain elevated across all the groups, indicating persistent anti-TPO positivity. The presence of high outliers in multiple age ranges points to intensified autoimmune responses in specific patients.

This box plot depicts the distribution of anti-thyroglobulin (anti-TG) antibody levels across age groups. Marked variability is observed among younger individuals (10–20 years), with a broad interquartile range and extreme outliers, suggesting diverse and heightened autoimmune activity. As age increases, the IQR progressively narrows, and median values decline, indicating a potential reduction in both anti-TG levels and autoimmune reactivity over time. The presence of high outliers, especially in younger groups, points to elevated autoimmune thyroid responses in specific cases.

### 2.3. Distribution of Anti-RBD Antibodies by Sex in Autoimmune Thyroiditis Patients

**Figure 4:**
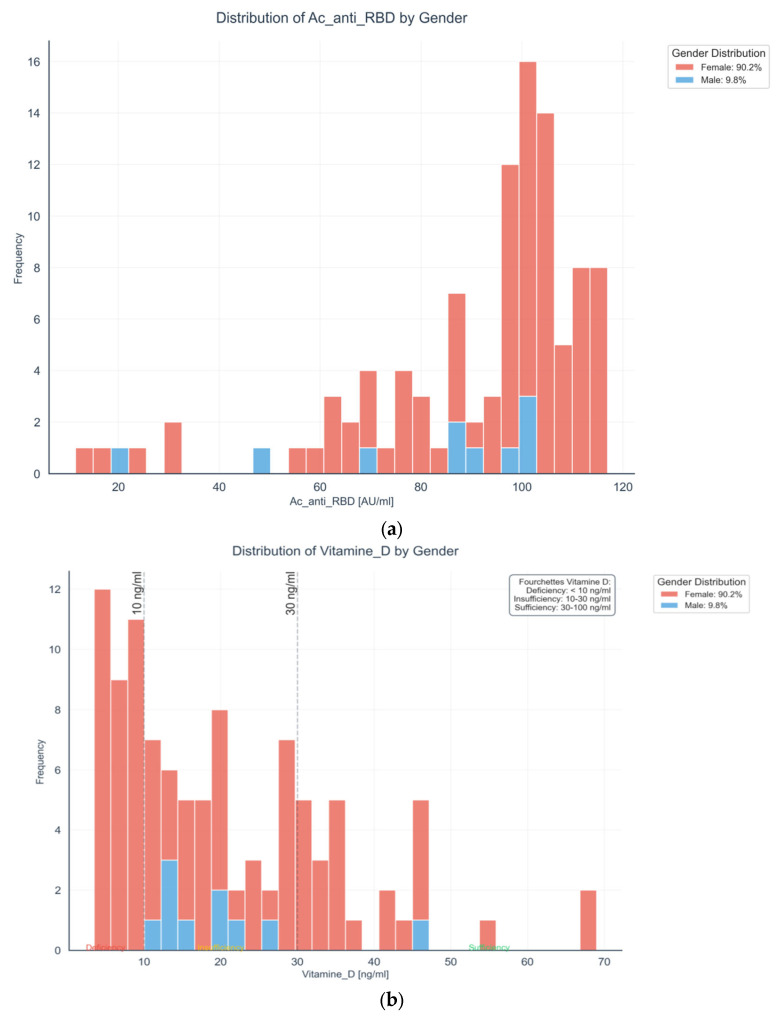

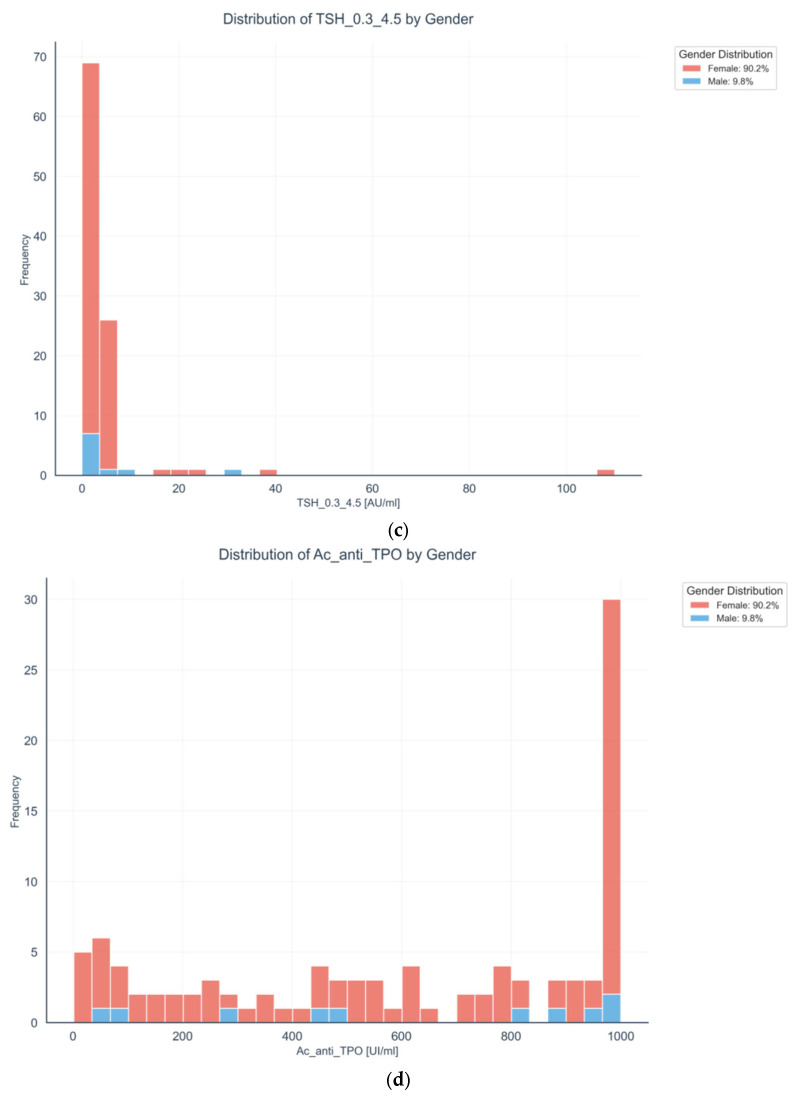

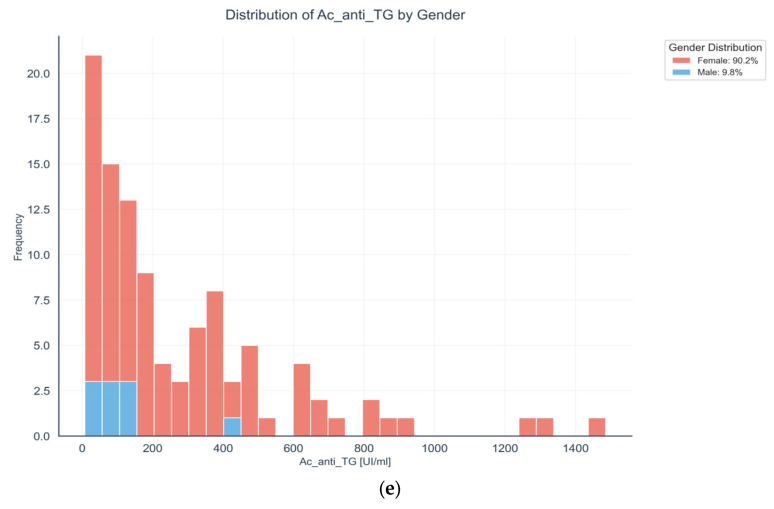
(**a**) Distribution of Anti-RBD Antibodies by Sex in Autoimmune Thyroiditis Patients. (**b**) Distribution of 25(OH) D by Sex in Autoimmune Thyroiditis Patients. (**c**) Distribution of TSH by Sex in Autoimmune Thyroiditis Patients. (**d**) Distribution of Anti-TPO by Sex in Autoimmune Thyroiditis Patients. (**e**) Distribution of Anti-TG Markers by Sex in Autoimmune Thyroiditis Patients. Abbreviations: Ac_anti_TG, Anti Thyroglobulin Antibodies; IU/mL, International Units per milliliter.

To explore sex-related differences in immune and endocrine profiles, we compared the distribution of key biomarkers between male and female patients with autoimmune thyroiditis following COVID-19 vaccination. As shown in Figure 4a, anti-RBD IgG antibody levels varied by sex, suggesting potential sex-based differences in humoral immune response. Figure 4b presents serum 25(OH) D levels, revealing disparities in vitamin D status between groups. In Figure 4c, TSH levels are compared across sexes, while Figure 4d,e illustrate the distributions of anti-TPO and anti-TG autoantibodies, respectively. These analyses provide insight into how biological sex may modulate both immune reactivity and thyroid-related parameters in this patient population.

Histogram compares anti-receptor binding domain (anti-RBD) IgG antibody levels between male and female patients with autoimmune thyroiditis. Female participants, who represent the vast majority of the cohort (90.2%), exhibit higher and more concentrated antibody titers around 100 AU/mL, indicating a robust humoral response post-COVID-19 vaccination. In contrast, male participants (9.8%) display a more variable distribution. Although limited by the smaller male sample size, these observations suggest that sex-related immunological and hormonal factors may influence vaccine-induced antibody responses.

This histogram illustrates serum 25(OH) vitamin D concentrations across sexes in patients with autoimmune thyroiditis. A predominant vitamin D deficiency (<10 ng/mL) is observed in both sexes, with a higher frequency among females, who represent 90.2% of the cohort. Most individuals exhibit levels below the sufficiency threshold (≥30 ng/mL), indicating widespread insufficiency. These findings may reflect sex-related differences in vitamin D metabolism, lifestyle, or environmental exposure influencing vitamin D status.

The distribution of TSH levels is highly skewed, with most patients exhibiting low to moderate TSH values (0–5 AU/mL). A small subset of individuals presents abnormally high TSH levels, reflecting severe thyroid dysfunction in certain cases. Females (90.2%) dominate the cohort, in line with the higher prevalence of autoimmune thyroid diseases in women. The presence of extreme outliers (>100 AU/mL) suggests that some patients experience severe thyroid dysregulation, possibly linked to post-COVID-19 immune disturbances or chronic autoimmune activity. The similar distribution patterns in both sexes indicate that while women are more frequently affected, the severity of thyroid dysfunction is comparable between sexes.

Histogram showing the distribution of anti-thyroid peroxidase (anti-TPO) antibody levels by sex in patients with autoimmune thyroiditis. A clear predominance of elevated anti-TPO levels was observed among females (90.2%), with a substantial proportion reaching values close to 1000 IU/mL. This pattern suggests a more pronounced autoimmune response in women, consistent with known sex-based differences in the prevalence of autoimmune thyroid disorders. Male participants exhibited lower frequencies and generally lower anti-TPO titers.

Histogram illustrating the distribution of anti-thyroglobulin (anti-TG) antibody levels by sex in patients with autoimmune thyroiditis. Female participants (90.2%) showed a higher prevalence and concentration of anti-TG antibodies compared to males. Most individuals had anti-TG levels below 400 IU/mL, though a few exceeded 1000 IU/mL, indicating marked thyroid autoimmunity in select cases. This pattern is consistent with the known higher susceptibility of women to autoimmune thyroid diseases.

### 2.4. In Silico Network Pharmacology and Molecular Docking Analysis to Explore the Mechanistic Effect of Vitamin D_3_ on Autoimmune Thyroiditis

#### Identification of Autoimmune Thyroiditis and Vitamin D_3_-Associated Targets

**Figure 5:**
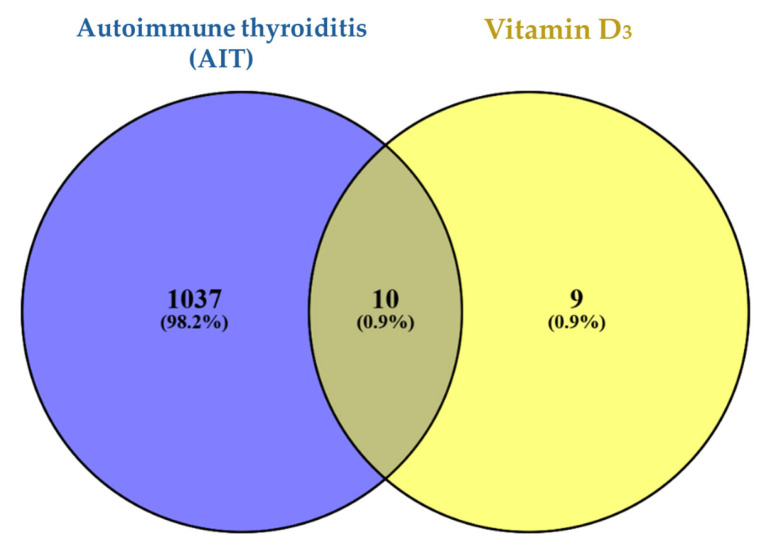
Venn diagram showing the intersections of Vitamin D and AIT-related genes.

A total of 1047 genes associated with autoimmune thyroiditis (AIT) were identified from GeneCards after filtering based on GIFTS score ≥60 to ensure the inclusion of high-confidence disease-related genes. In parallel, 19 vitamin D_3_-related targets were collected from the SwissTargetPrediction platform (https://www.swisstargetprediction.ch/, accessed on 11 November 2025). By comparing these two gene sets using a Venn diagram, 10 overlapping targets were identified, representing the potential molecular interface through which vitamin D_3_ may exert regulatory effects on autoimmune thyroiditis (Figure 5).

The limited number of overlapping targets reflects the use of stringent probability thresholds in SwissTargetPrediction, prioritizing high-confidence vitamin D_3_ interactions. This approach was intentionally adopted to reduce false-positive associations and to focus subsequent analyses on the most reliable candidate targets.

### 2.5. Construction of the PPI Network and Identification of Hub Genes

**Figure 6:**
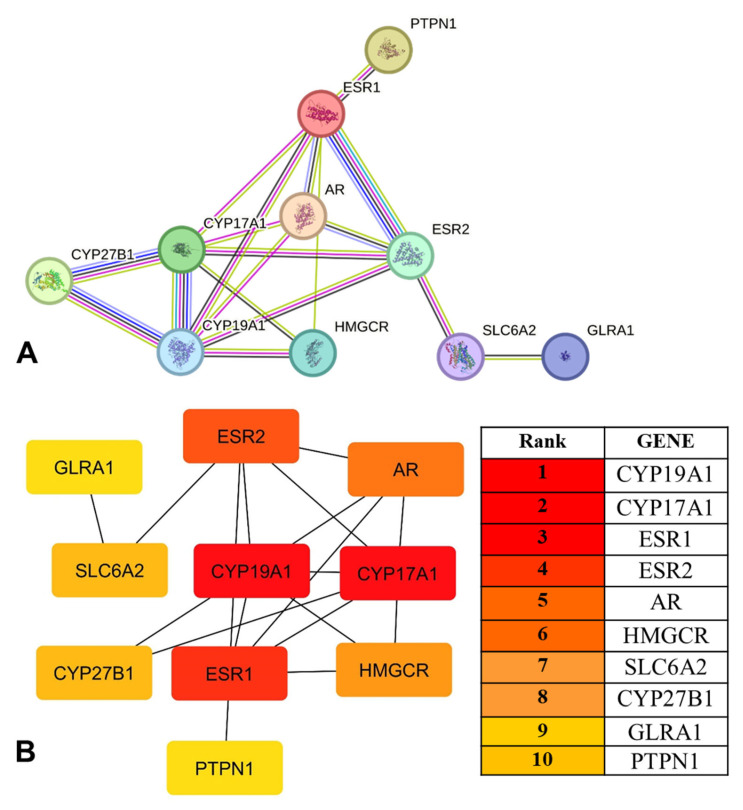
(**A**) Protein–protein interaction (PPI) network of shared vitamin D_3_ and autoimmune thyroiditis targets. (**A**) Overall PPI network was constructed using STRING. (**B**) Hub gene identification based on topological analysis using the cytoHubba the Maximal Clique Centrality (MCC) algorithm in Cytoscape. Node size and color intensity reflect relative interaction degree, with CYP19A1, CYP17A1, and ESR1 highlighted as the most connected nodes within the network.

The overlapping genes were imported into the STRING database using a medium-confidence interaction score (≥0.4), resulting in a protein–protein interaction (PPI) network composed of 10 nodes and 18 edges. Despite the limited size of the network, a connected interaction pattern was observed, indicating non-random biological relationships among the shared targets. Network visualization and topological analysis were performed using Cytoscape. Node importance was evaluated based on degree, betweenness, and closeness centrality metrics. Hub gene prioritization was conducted using the cytoHubba plugin, applying the Maximal Clique Centrality (MCC) algorithm. Based on this analysis, the top-ranked genes included CYP19A1, CYP17A1, ESR1, ESR2, AR, HMGCR, SLC6A2, CYP27B1, GLRA1, and PTPN1 (Figure 6). These genes are primarily involved in endocrine and steroidogenic regulation rather than immune-specific. Extensive evidence indicates that steroidogenic enzymes and nuclear hormone receptors exert indirect regulatory effects on humoral immunity through modulation of B-cell responses, cytokine networks, and immune tolerance, particularly in autoimmune and vaccine-responsive contexts.

Considering the modest size of the overlapping target set, these highly connected genes are represented as central nodes within a conservative, high-confidence interaction framework. However, the presence of steroidogenic enzymes and nuclear hormone receptors among these hubs is significant. It suggests that endocrine regulatory pathways are a key interface linking vitamin D_3_ signaling to autoimmune thyroid dysfunction.

### 2.6. Functional Enrichment Analysis

Functional enrichment analysis of the identified hub genes was performed to explore biological processes and pathways potentially linking vitamin D_3_ signaling and autoimmune thyroiditis. Given the limited gene set, enrichment results were interpreted as exploratory. KEGG pathway analysis revealed significant enrichment of pathways related to steroid biosynthesis, hormone signaling, and endocrine regulation (Table 1). CYP27B1 was associated with steroid biosynthesis (FDR = 0.023), while HMGCR mapped to terpenoid backbone biosynthesis (FDR = 0.025), reflecting their roles in vitamin D_3_ metabolism and cholesterol-derived hormone synthesis.

Several sex hormone-related pathways were prominently enriched. The prolactin signaling pathway (FDR = 9.6 × 10^−5^) included CYP17A1, ESR1, and ESR2, while ovarian steroidogenesis (FDR = 0.002) and steroid hormone biosynthesis (FDR = 0.002) were represented by CYP17A1 and CYP19A1.

Additionally, estrogen signaling and endocrine resistance pathways (FDR < 0.01) involved ESR1 and ESR2, both of which have been reported to modulate immune cell activation, cytokine production, and antibody responses through hormone-dependent mechanisms.

**Figure 7:**
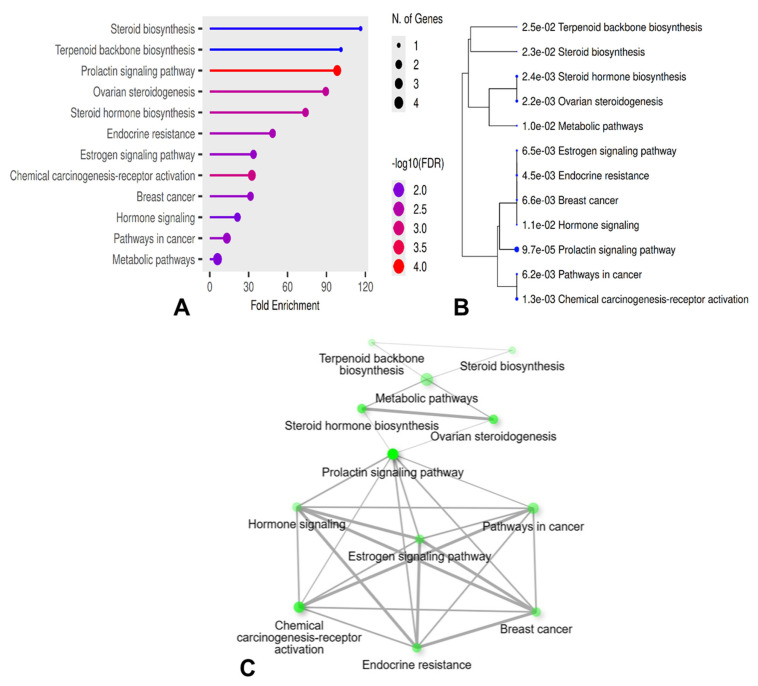
Functional enrichment analysis of top-ranked genes associated with vitamin D_3_ and autoimmune thyroiditis (**A**) Kyoto Encyclopedia of Genes and Genomes (KEGG) pathway enrichment bar plot. (**B**) Hierarchical clustering dendrogram of enriched pathways. (**C**) Pathway-gene interaction network illustrating the relationships between hub genes and enriched endocrine and metabolic pathways.

Cancer and cancer metabolism-related pathways, including chemical carcinogenesis–receptor activation and general metabolic pathways, were also enriched, reflecting the broad transcriptional and regulatory functions of nuclear hormone receptors and steroidogenic enzymes. Collectively, these enrichment patterns suggest that the vitamin D_3_–AIT interaction network is predominantly driven by endocrine and metabolic pathways with known immunomodulatory potential. (Figure 7).

### 2.7. Molecular Docking Results

Molecular docking was performed to explore the structural compatibility between vitamin D_3_ (cholecalciferol) and three key targets identified through network pharmacology analysis, CYP19A1 (PDB: 3S79), CYP17A1 (PDB: 3RUK), and ESR1 (PDB: 1XP1). These proteins were selected based on their central roles in steroid hormone biosynthesis and estrogen signaling pathways with known immunomodulatory potential. Docking protocol reliability was assessed through redocking of the native co-crystallized ligands into their respective binding sites. The redocked poses reproduced favorable Glide XP scores for androstenedione in CYP19A1 (−8.234 kcal/mol), abiraterone in CYP17A1 (−8.752 kcal/mol), and the native ligand (AIH) in ESR1 (−7.406 kcal/mol), supporting the reliability of the docking setup (Table 2).

To further evaluate pose accuracy, the docked vitamin D_3_ conformations were structurally aligned with the corresponding native ligands, and root-mean-square deviation (RMSD) values were calculated (Table 2). The resulting RMSD values were 2.146 Å for CYP19A1, 1.953 Å for CYP17A1, and 0.007 Å for ESR1. These RMSD values were within an acceptable range (˂2 Å), indicating acceptable pose overlap for the cytochrome P450 enzymes and near-identical positioning within the ESR1 ligand-binding domain.

**Figure 8:**
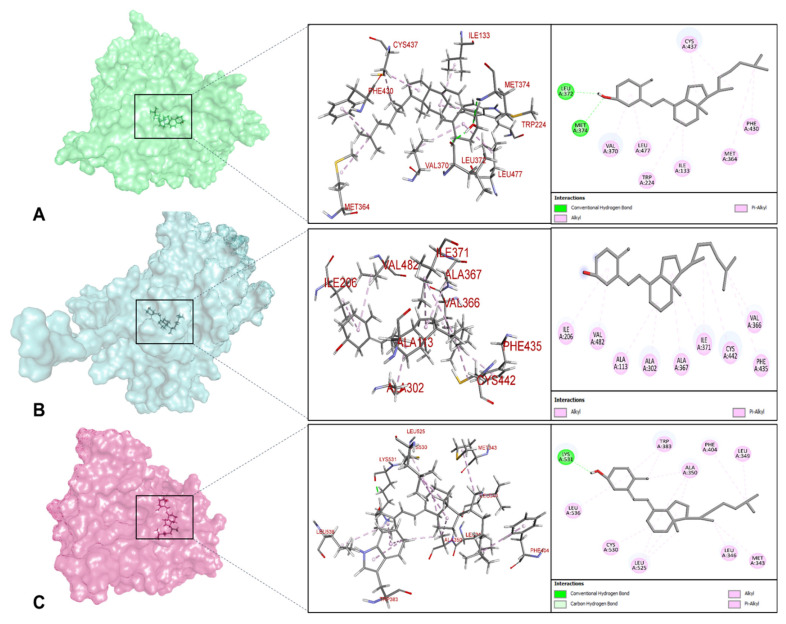
Molecular docking 3D and 2D visualization of vitamin D_3_ bound to the active sites of (**A**) CYP19A1 (PDB: 3S79), (**B**) CYP17A1 (PDB: 3RUK), and (**C**) ESR1 (PDB: 1XP1).

Vitamin D_3_ demonstrated favorable binding affinities across all three targets. For CYP19A1, a docking score of −7.382 kcal/mol was obtained, indicating moderate affinity within the aromatase active site. The binding pose revealed two hydrogen bonds with LEU372 and MET374 (2.01–2.52 Å), along with eight hydrophobic interactions involving ILE133, TRP224, MET364, VAL370, PHE430, CYS437, and LEU477 (4.45–5.40 Å) (Figure 8A). These interactions positioned vitamin D_3_ within the steroid-binding cavity, suggesting structural compatibility with the enzyme’s pocket.

In CYP17A1, vitamin D_3_ achieved a docking score of −7.289 kcal/mol. Although hydrogen bonds were not detected, the ligand was stabilized by ten hydrophobic contacts with residues ALA113, ILE206, ALA302, VAL366, ALA367, ILE371, PHE435, CYS442, and VAL482 (3.49–5.43 Å) (Table 3; Figure 8B). This interaction profile is consistent with the highly lipophilic nature of the CYP17A1 active site.

The strongest interaction was observed for ESR1, where vitamin D_3_ yielded a docking score of -8.016 kcal/mol, exceeding that of the native ligand. Two hydrogen bonds with LYS531 (1.99–2.89 Å) were observed, accompanied by extensive hydrophobic interactions involving residues ALA350, LEU525, CYS530, LEU536, MET343, LEU346, LEU349, TRP383, and PHE404 (3.31–5.44 Å) (Table 3; Figure 8C). The RMSD value further confirmed a highly stable and well-defined binding orientation within the estrogen receptor ligand-binding domain.

The obtained docking results indicated that vitamin D_3_ could be structurally accommodated within the binding sites of CYP19A1, CYP17A1, and ESR1, supporting potential interaction compatibility with endocrine regulators implicated in autoimmune thyroiditis.

### 2.8. Molecular Dynamics Simulations Results

**Figure 9:**
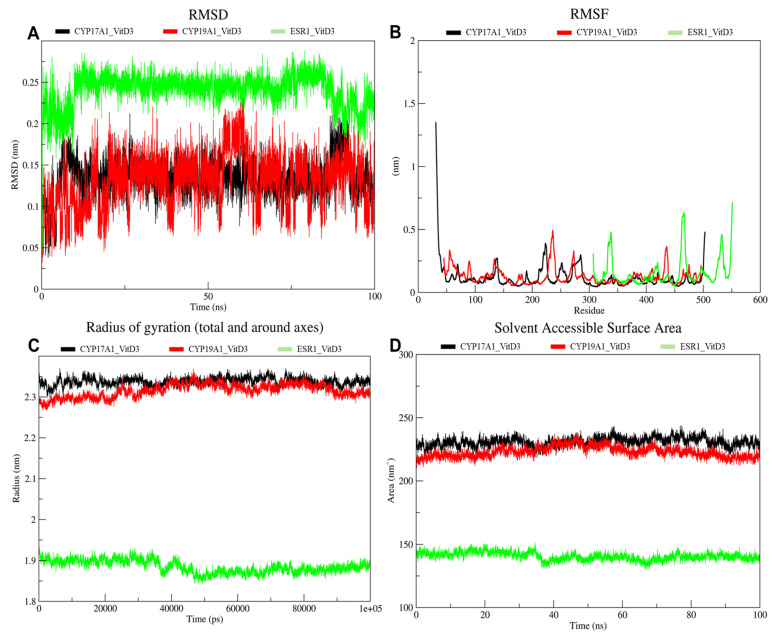
Molecular dynamics simulation analyses of vitamin D_3_ complexes with CYP17A1, CYP19A1, and ESR1 over 100 ns. (**A**) root mean square deviation (RMSD) as a function of simulation time, comparing the conformational stability of the CYP17A1-Vitamin D_3_ (black), CYP19A1-Vitamin D_3_ (red), and ESR1-Vitamin D_3_ (green) complexes. (**B**) Root mean square fluctuation (RMSF) per residue, illustrating residue-wise flexibility profiles for the three complexes. (**C**) Radius of gyration (Rg) plotted over time, reflecting the overall compactness of each protein–vitamin D_3_ complex during the simulation. (**D**) Solvent-accessible surface area (SASA) as a function of time, indicating changes in protein surface exposure upon vitamin D_3_ binding.

The three vitamin D3 protein complexes were further evaluated through molecular dynamics (MD) simulations to assess their structural stability and conformational behavior. During the 100 ns MD simulations, all of the complexes exhibited stable root mean square deviation (RMSD) profiles after an initial equilibration phase (Figure 9A). The CYP19A1-Vitamin D_3_ and CYP17A1-Vitamin D_3_ complexes showed relatively low RMSD values throughout the simulation, with fluctuations remaining within a narrow range after the initial equilibration period. The calculated average RMSD values were 0.130 nm for CYP19A1-Vitamin D_3_ and 0.135 nm for CYP17A1-Vitamin D_3_ (Table 4). In contrast, the ESR1-Vitamin D_3_ complex exhibited higher RMSD values over the simulation time, with more pronounced fluctuations compared to the cytochrome P450 complexes. The average RMSD value for ESR1-Vitamin D_3_ was 0.241 nm. Despite these differences, all three complexes maintained RMSD values below 0.3 nm across the simulation period, indicating stable trajectories without major structural deviations.

The root mean square fluctuation (RMSF) was used to assess residue-level flexibility of the three vitamin D_3_ complexes (Figure 9B). For all the complexes, the majority of residues exhibited low fluctuation values, with RMSF values generally remaining below 0.3 nm, indicating limited backbone mobility across most of the protein structures. The CYP17A1-Vitamin D_3_ complex showed the lowest overall fluctuations, with an average RMSF value of 0.114 nm, while CYP19A1-Vitamin D_3_ displayed slightly higher residue mobility, with an average RMSF of 0.126 nm. The ESR1-Vitamin D_3_ complex exhibited comparatively higher fluctuations, particularly in specific residue regions, resulting in a higher average RMSF value of 0.157 nm. Notable localized peaks were observed in all three complexes, corresponding to flexible loop or terminal regions, whereas the remaining residues maintained relatively stable fluctuation profiles throughout the simulations.

The compactness of the protein vitamin D_3_ complexes during the 100 ns molecular dynamics simulations was evaluated by monitoring the radius of gyration (Rg) as a function of time (Figure 9C). The CYP17A1-Vitamin D_3_ and CYP19A1-Vitamin D_3_ complexes exhibited relatively stable Rg profiles throughout the simulation, with minor fluctuations around their respective mean values. The average Rg was 2.337 nm for CYP17A1-Vitamin D_3_ and 2.315 nm for CYP19A1-Vitamin D_3_, indicating that both complexes maintained a consistent overall structural compactness over time. In contrast, the ESR1- Vitamin D_3_ complex displayed lower Rg values across the simulation, with an average Rg of 1.885 nm. A slight decrease in Rg was observed during the mid-simulation period, followed by stabilization toward the end of the trajectory. The Rg profiles suggest that all three complexes preserved their global folding during the simulation, with no pronounced structural expansion or collapse observed.

The solvent accessible surface area (SASA) of the protein with vitamin D_3_ complexes was monitored over the 100 ns molecular dynamics simulations to assess changes in solvent exposure (Figure 9D). The CYP17A1-Vitamin D_3_ complex exhibited consistently higher SASA values throughout the trajectory, fluctuating around an average of 230.901 nm^2^, with moderate temporal variations but no abrupt shifts. Similarly, the CYP19A1-Vitamin D_3_ complex showed a stable SASA profile, with values centered around an average of 222.974 nm^2^, displaying small fluctuations over time. In contrast, the ESR1-Vitamin D_3_ complex presented markedly lower SASA values compared to the cytochrome P450 complexes, with an average SASA of 140.650 nm^2^. The results indicate that solvent exposure of all three complexes remained relatively consistent during the simulation timeframe, with no pronounced or sustained changes observed.

**Figure 10:**
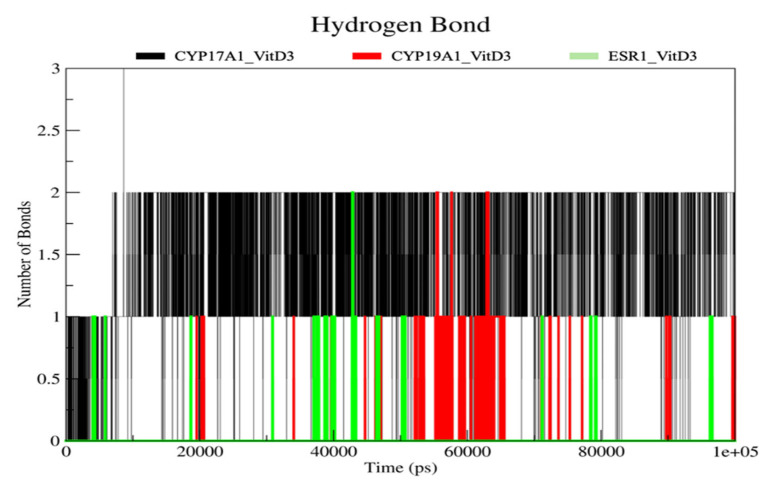
Number of intermolecular hydrogen bonds formed between vitamin D_3_ and each protein (CYP17A1, CYP19A1, and ESR1) throughout the simulation period.

The number of hydrogen bonds formed between vitamin D_3_ and the target proteins was monitored throughout the molecular dynamics simulations (Figure 10). For the CYP17A1-Vitamin D_3_ complex, hydrogen bonds were observed intermittently over the simulation time, with the number of hydrogen bonds fluctuating mainly between 0 and 2, and occasional persistence of 1–2 hydrogen bonds during extended periods of the trajectory. Similarly, the CYP19A1-Vitamin D_3_ complex displayed transient hydrogen bond formation, predominantly ranging from 0 to 2 hydrogen bonds, with several intervals showing the presence of one hydrogen bond and sporadic occurrences of two hydrogen bonds, particularly during the mid-simulation phase. These interactions were not continuous but appeared repeatedly across the simulation time. In contrast, the ESR1-Vitamin D_3_ complex exhibited fewer hydrogen bonds overall. The hydrogen bond count for this complex remained predominantly at 0 or 1, with occasional short-lived appearances of up to 2 hydrogen bonds, indicating more limited and transient hydrogen bond interactions compared to the cytochrome P450 complexes. The hydrogen bond analysis suggests that vitamin D_3_ engages in intermittent and dynamic hydrogen bonding interactions with all three proteins during the simulation period, without the presence of a continuously maintained hydrogen bond network.

**Figure 11:**
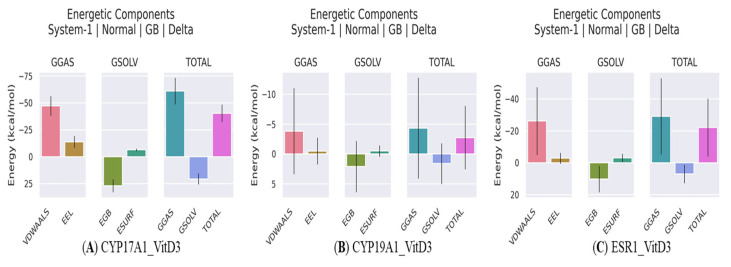
Molecular Mechanics/Generalized Born Surface Area (MM/GBSA) energetic components for Vitamin D3 binding to (**A**) CYP17A1, (**B**) CYP19A1, and (**C**). The free energy contributions are decomposed into van der Waals (VDWAALS) and electrostatic (EEL) gas-phase interactions (GGAS), polar solvation energy (EGB) and non-polar solvation energy (ESURF) (GSOLV), and the resulting total binding free energy (TOTAL).

MM-GBSA calculations were performed to estimate the binding free energies and energetic contributions of vitamin D_3_ toward CYP19A1, CYP17A1, and ESR1 (Table 5 and Figure 11). The CYP17A1-vitamin D_3_ complex showed the most favorable binding free energy, with a total ΔG of −40.4 kcal/mol. This interaction was primarily driven by strong van der Waals (VDWAALS = −47.27 kcal/mol) and electrostatic (EEL = −13.73 kcal/mol) contributions, resulting in a highly favorable gas-phase energy (GGAS = −61.0 kcal/mol). These interactions were partially counterbalanced by polar solvation effects (EGB = 26.96 kcal/mol), yielding an overall negative binding free energy.

The ESR1-vitamin D_3_ complex also exhibited a favorable binding profile, with a total ΔG of −22.15 kcal/mol. Similar to CYP17A1, van der Waals interactions (−26.23 kcal/mol) represented the dominant stabilizing force, accompanied by modest electrostatic contributions (−2.89 kcal/mol). Although polar solvation energy opposed binding (EGB = 10.12 kcal/mol), the overall gas-phase contribution (GGAS = −29.12 kcal/mol) remained sufficient to support a net favorable interaction. In contrast, the CYP19A1–vitamin D_3_ complex displayed a substantially weaker binding affinity, with a total ΔG of −2.75 kcal/mol. Both van der Waals (−3.84 kcal/mol) and electrostatic (−0.50 kcal/mol) contributions were limited, and the stabilizing effects were largely offset by solvation energies, resulting in only a marginally favorable binding free energy. The obtained MM-GBSA results indicate differential binding strengths of vitamin D_3_ across the three targets, with stronger predicted interactions for CYP17A1 and ESR1, and comparatively weaker binding for CYP19A1 under the applied simulation conditions.

## 3. Discussion

The present study examined the relationship between vitamin D status and humoral responses to SARS-CoV-2 vaccination in patients with autoimmune thyroiditis, a population characterized by altered endocrine–immune regulation. An inverse association was observed between serum 25-hydroxyvitamin D levels and anti–SARS-CoV-2 spike RBD IgG titers, indicating that lower vitamin D status was associated with higher post-vaccination antibody concentrations. Given the cross-sectional design, this finding reflects an association at a single time point and should not be interpreted as evidence of causality. Importantly, the magnitude of antibody responses does not necessarily equate to optimal or protective immunity. In autoimmune-prone individuals, elevated humoral responses may reflect altered immune regulation rather than enhanced vaccine efficacy. Within this context, heightened antibody production may represent a state of increased immune reactivity, potentially influenced by underlying autoimmune activity and endocrine imbalance. These observations are consistent with existing evidence suggesting that vitamin D plays a regulatory role in immune homeostasis by influencing T- and B-cell differentiation, cytokine signaling, and inflammatory responses [20,21,22].

In patients with autoimmune thyroiditis, particularly Hashimoto’s thyroiditis, vitamin D deficiency has been linked to increased Th1 and Th17 activity, fostering a pro-inflammatory environment that may exacerbate autoimmune processes [18]. Consistent with this, our analysis of vitamin D distribution by age group revealed widespread insufficiency, with a large proportion of patients falling below the 30 ng/mL sufficiency threshold, further supporting the hypothesis that hypovitaminosis D contributes to immune dysregulation in this population.

Patients with autoimmune thyroiditis who were vitamin D-deficient exhibited significantly higher anti-SARS-CoV-2 RBD antibody titers (mean: 105.6 AU/mL) compared to those with sufficient vitamin D levels (mean: 78.4 AU/mL), suggesting the presence of a dysregulated humoral immune response. Several mechanisms may underlie this observation: first, impaired regulatory T cell (Treg) function in the context of vitamin D deficiency may contribute to reduced immune control; second, increased levels of pro-inflammatory cytokines such as IL-6, TNF-α, and IL-17—normally downregulated by vitamin D—may amplify immune activation; and third, enhanced B-cell activity may be reflected in elevated anti-TPO and anti-TG antibody levels, particularly among female patients. These results suggest that vitamin D deficiency could amplify antibody production following vaccination, potentially heightening autoimmune reactivity rather than supporting a balanced and protective immune outcome [23].

The marked female predominance in our study cohort (90.2%) aligns with well-established epidemiological trends in autoimmune thyroiditis, where women are disproportionately affected. This sex disparity is attributed to several biological mechanisms. Hormonal influences play a central role: estrogen enhances B-cell activation and autoantibody production, whereas testosterone exerts immunosuppressive effects [24]. Genetic predisposition also contributes, notably through X-linked immune-related genes such as *Toll-like receptor 7* (TLR7), which are more highly expressed in females and promote robust innate immune responses [25]. Additionally, women generally exhibit stronger immune reactions to both infections and vaccines, potentially contributing to the greater variability in antibody titers observed in our cohort. Histograms illustrating anti-TPO and anti-TG antibody distributions by sex further reinforce this trend, showing markedly elevated levels among female participants. These findings support the role of sex hormones and genetic architecture in shaping autoimmune susceptibility and immune dysregulation.

The inverse relationship between vitamin D status and post-vaccination antibody concentrations highlights important clinical and immunological implications. Patients with sufficient vitamin D levels (≥30 ng/mL) demonstrated more regulated humoral responses, likely reflecting vitamin D’s capacity to modulate B-cell function by inhibiting plasma cell differentiation and limiting excessive antibody production [25]. Additionally, vitamin D promotes the secretion of anti-inflammatory cytokines such as IL-10 and TGF-β, while suppressing pro-inflammatory mediators including IL-6, IL-17, and TNF-α. It also favors the expansion of regulatory T cells over pro-inflammatory Th1 and Th17 subsets, thereby reducing the risk of autoimmune flare-ups.

**Figure 12:**
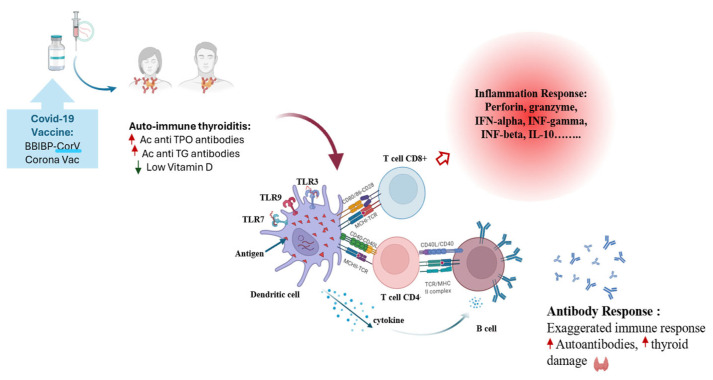
Immunological impact of vitamin D deficiency on vaccine-induced antibody responses.

The high variability in antibody responses observed among vitamin D-deficient individuals suggests that optimizing vitamin D status prior to vaccination may help stabilize immune reactivity and potentially reduce the risk of vaccine-related autoimmune complications [16,25]. To illustrate these mechanisms, Figure 12 provides a schematic overview of how low vitamin D levels may contribute to dysregulated immune activation and excessive antibody production following COVID-19 vaccination in patients with autoimmune thyroiditis.

This schematic illustrates the potential effects of low vitamin D status on immune regulation in the context of COVID-19 vaccination. In vitamin D-deficient individuals, reduced secretion of anti-inflammatory cytokines (IL-10, TGF-β) combined with increased production of pro-inflammatory mediators (IL-6, IL-17, TNF-α) promotes Th1 and Th17 polarization and enhances B-cell activation. These immune shifts may lead to exaggerated anti-RBD IgG responses and increased thyroid autoantibody levels, particularly in individuals predisposed to autoimmune disease.

Selenium has also emerged as a potential modulator of autoimmune thyroid disease due to its critical role in antioxidant defense and immune regulation. It contributes to thyroid hormone synthesis and metabolism by reducing reactive oxygen species and maintaining redox balance. In addition, selenium supports regulatory T cell activity and may help suppress the production of thyroid autoantibodies [26]. Although current evidence remains preliminary, co-supplementation strategies involving both vitamin D and selenium during vaccination have been proposed to enhance immune regulation via synergistic antioxidant and immunomodulatory effects. Further research is needed to clarify the interplay between selenium, vitamin D, oxidative stress, and immune homeostasis in the context of thyroid autoimmunity and vaccine-induced immune responses [27,28].

Despite the strength of these findings, several limitations should be acknowledged. This study was conducted on a moderately sized cohort using a cross-sectional design, which may limit the generalizability of the results. Additionally, vitamin D levels and antibody titers were measured at a single time point, precluding assessment of dynamic immune changes over time. Potential confounders such as sun exposure, dietary intake, supplementation compliance, and comorbidities were not fully controlled. Future multicenter longitudinal studies are warranted to validate these results and elucidate the underlying causal mechanisms.

The network pharmacology analysis yielded a modest overlap between vitamin D_3_-associated targets and autoimmune thyroiditis-related genes, reflecting the application of conservative, high-confidence filtering criteria rather than an absence of biological relevance. Accordingly, downstream protein–protein interaction, enrichment, and docking analyses were interpreted as exploratory and hypothesis-generating rather than mechanistically definitive.

Within this framework, enrichment analysis of the prioritized top-ranked genes (CYP19A1, CYP17A1, ESR1, ESR2, AR, HMGCR, CYP27B1, SLC6A2, GLRA1, and PTPN1) highlighted pathways related to steroid biosynthesis, estrogen and prolactin signaling, hormone signaling, and metabolic regulation. These pathways are increasingly recognized as indirect modulators of immune function, as dysregulated sex-steroid and endocrine signaling can influence immune tolerance, T-cell polarization, and autoantibody production in autoimmune disorders. Notably, ESR1 and ESR2, central components of estrogen signaling, were consistently enriched, supporting the established role of sex hormones in shaping autoimmune susceptibility, particularly in thyroid disease. Similarly, the identification of steroidogenic enzymes such as CYP17A1 and CYP19A1 suggests that vitamin D_3_ signaling may intersect with broader hormonal regulatory networks rather than acting solely through classical immune pathways [27].

The inclusion of CYP27B1, responsible for local activation of vitamin D within peripheral tissues, further reinforces the biological plausibility of crosstalk between vitamin D metabolism and endocrine–immune regulation [29]. Collectively, these findings support a conceptual model in which vitamin D deficiency may contribute to autoimmune thyroid dysfunction through indirect modulation of endocrine pathways that influence immune reactivity, while acknowledging that experimental validation is required to confirm these relationships.

Molecular docking analyses were performed to structurally explore the interactions suggested by the network pharmacology results. Vitamin D_3_ was accommodated within the binding pockets of CYP17A1, CYP19A1, and ESR1, with docking poses indicating favorable spatial complementarity. Pose validation based on RMSD values supported the reliability of the predicted binding orientations, particularly for ESR1, where vitamin D_3_ adopted a conformation closely aligned with the native ligand. Given the established involvement of estrogen receptor signaling in female-predominant autoimmune disorders and humoral immune regulation, the interaction with ESR1 provides a plausible structural link between vitamin D_3_ and endocrine pathways relevant to autoimmune thyroid disease [27,30]. The compatibility of vitamin D_3_ with CYP17A1 and CYP19A1 further suggests potential intersections with steroidogenic pathways that regulate hormone availability and downstream immune responses [31]. However, these docking results represent structural binding compatibility rather than evidence of functional modulation.

To further evaluate the stability of the docked complexes under dynamic conditions, molecular dynamics (MD) simulations were conducted. MD simulations assess the temporal behavior of ligand-protein complexes in a solvated environment, allowing verification of whether the predicted poses remain stable over time. Across all the simulated systems, the vitamin D_3_-protein complexes remained structurally stable, with no indications of major conformational disruption or ligand dissociation during the simulation period.

Backbone deviation and fluctuation analyses indicated that the proteins preserved their overall structural integrity, with flexibility mainly restricted to peripheral or solvent-exposed regions, while residues surrounding the binding sites showed relatively limited motion. Global structural descriptors further suggested maintenance of protein compactness and solvent accessibility throughout the simulations, indicating that vitamin D_3_ binding did not induce unfavorable conformational changes.

Hydrogen bond analysis revealed transient and intermittent interactions rather than persistent hydrogen bond networks, suggesting that the stabilization of the complexes was largely driven by hydrophobic and van der Waals contributions [29]. This observation is consistent with the physicochemical properties of vitamin D_3_ and the predominantly nonpolar nature of the identified binding pockets. MM-GBSA analysis provided complementary energetic insight, indicating favorable binding energetics dominated by gas-phase interaction components, partially offset by solvation effects. As noted in this study, these estimates should be interpreted qualitatively and used for comparative assessment rather than as absolute binding free energies.

The MD analysis supports the structural and dynamic stability of the vitamin D_3_ binding modes identified by docking. The MD results are presented as supportive evidence in which vitamin D deficiency may contribute to altered endocrine–immune crosstalk. Together with the network pharmacology and docking analyses, these findings suggest that vitamin D_3_ may stably associate with key endocrine-related targets implicated in autoimmune thyroid disease, requiring further experimental investigation to clarify biological relevance.

The present findings suggest that vitamin D status may represent a modifiable factor influencing vaccine-induced immune responses in patients with autoimmune thyroiditis. The results suggest that optimizing vitamin D levels could potentially contribute to more balanced immune reactivity in autoimmune populations. Future studies should aim to define optimal vitamin D thresholds that support immune regulation without promoting excessive humoral activation. In this context, the immunomodulatory role of selenium also requires further investigation, given its involvement in antioxidant defense, thyroid hormone metabolism, and immune regulation. Longitudinal studies assessing the combined effects of vitamin D and selenium on thyroid function, autoantibody production, and immune responses following vaccination would provide valuable mechanistic and clinical insights. A multidisciplinary research approach involving endocrinology, immunology, and infectious disease expertise will be essential to refine preventive strategies. In addition, lifestyle-related factors such as nutrition, physical activity, and stress management should be explored as potential modifiers of micronutrient–immune interactions.

Overall, this study highlights the complex interplay between vitamin D status, vaccine-induced antibody responses, and thyroid autoimmunity. The observed inverse association between serum vitamin D levels and anti-RBD IgG titers suggests that insufficient vitamin D may be associated with heightened and potentially dysregulated humoral immune responses in AIT. The pronounced female predominance and elevated thyroid autoantibody levels are consistent with established sex-related differences in immune regulation and autoimmune susceptibility. Together, these findings support the relevance of considering endocrine–immune interactions when evaluating vaccine responses in autoimmune conditions. While causality cannot be inferred from the present study, integrating vitamin D assessment into future pre-vaccination research frameworks may help clarify its role in optimizing immune responses while minimizing immune dysregulation in susceptible individuals.

## 4. Materials and Methods

### 4.1. Ethical Approval and Informed Consent

The study protocol was reviewed and approved by the Clinical Research Ethics Committee under reference code EC005MO12062022. The research was conducted in full compliance with national ethical regulations and the principles of the Declaration of Helsinki (2013 revision). All the participants were informed about the objectives of the study, the procedures involved, and their right to withdraw at any time without consequences. Written informed consent was obtained from all the participants prior to inclusion. Participant confidentiality and data anonymity were strictly maintained throughout the study.

### 4.2. Study Design, Area and Population

This cross-sectional observational study included 102 adult patients (≥18 years) diagnosed with autoimmune thyroiditis (AIT) in an Algerian region. During patient recruitment, information regarding current or recent use of vitamin D or multivitamin supplements was systematically collected through structured patient interviews. Individuals who reported active supplementation with vitamin D or other micronutrients known to influence immune or endocrine function were excluded from the study to minimize potential confounding effects on serum 25-hydroxyvitamin D levels and immune response measurements. Diagnosis of AIT was established based on clinical evaluation, elevated thyroid autoantibodies (anti-thyroid peroxidase [anti-TPO] and/or anti-thyroglobulin [anti-TG]), and thyroid function parameters. All the participants had received at least one dose of a SARS-CoV-2 vaccine prior to sample collection. The study was conducted during a four-month period following the implementation of national COVID-19 vaccination programs. The main objective was to evaluate the association between serum vitamin D levels and vaccine-induced humoral immune responses in patients with autoimmune thyroiditis.

Participants who tested positive for SARS-CoV-2 antibodies were considered seropositive due to previous infection and/or vaccination. Vaccines administered included non-replicating viral vector vaccines (Vaxzevria/AstraZeneca Cambridge, UK, Janssen/Johnson & Johnson New Brunswick, NJ, USA, and Sputnik V/Gamaleya Research Institute, Moscow, Russia) and inactivated vaccines (/CoronaVac /Sinovac Biotech, Beijing, China; Sinopharm/BBIBP-CorV Beijing, China). Blood samples (4 mL) were collected from the cubital vein using clot-activator tubes. After centrifugation, serum samples were aliquoted and stored at −20 °C until analysis.

This study did not include a healthy control group; therefore, analyses were restricted to within-cohort comparisons.

### 4.3. Measurement of Total Serum 25(OH) Vitamin D

Serum 25-hydroxyvitamin D [25(OH)D] concentrations were measured using a fully automated chemiluminescence immunoassay (CLIA) (MAGLUMI^®^, Shenzhen, China). reference no. 130211004M), according to the manufacturer’s instructions. Based on measured values, patients were classified as vitamin D deficient (<10 ng/mL), insufficient (10–29 ng/mL), or sufficient (≥30 ng/mL). The cutoff of <10 ng/mL was selected based on the manufacturer’s technical specifications.

### 4.4. Measurement of SARS-CoV-2 S-RBD IgG Antibodies

Quantitative detection of anti-SARS-CoV-2 spike receptor-binding domain (S-RBD) IgG antibodies was performed using an in vitro CLIA, according to the manufacturer’s protocol (Reference No. 130219017 M). Serum level values under 1.00 AU/mL were considered negative, and levels ≥1.00 AU/mL were considered positive. For patients with autoimmune thyroiditis, thyroid-stimulating hormone (TSH) and thyroid-specific autoantibodies (anti-TPO and anti-TG) were measured in the same laboratory using the same platform.

### 4.5. Measurement of Thyroid-Stimulating Hormone (TSH)

TSH levels were assessed using the MAGLUMI in vitro CLIA method (Reference No. 13020300M). The expected reference range was 0.3–4.5 µIU/mL. TSH is composed of two subunits, with the beta subunit conferring its biological and immunological specificity.

### 4.6. Measurement of Anti-Thyroid Peroxidase Antibodies (Anti-TPO)

Thyroid peroxidase (TPO) is a membrane-bound glycoprotein enzyme involved in thyroid hormone synthesis. Anti-TPO antibodies were quantitatively measured using the MAGLUMI CLIA method, following the manufacturer’s guidelines (Reference No. 130203011 M). The expected reference value was <30 IU/mL.

### 4.7. Measurement of Anti-Thyroglobulin Antibodies (ATG)

Anti-thyroglobulin antibodies were quantified in human serum using the MAGLUMI CLIA kit, according to the manufacturer’s instructions (Reference No. 130203007 M). The expected reference value was <95 IU/mL.

### 4.8. Network Pharmacology and Molecular Docking to Explore Therapeutic Targets and Signaling Mechanisms of Vitamin D_3_ in Autoimmune Thyroiditis

#### 4.8.1. Identification of Autoimmune Thyroiditis–Associated Targets

Autoimmune thyroiditis (AIT)-related genes were obtained from the GeneCards database (https://www.genecards.org/, accessed 2 November 2025) by using specified disease keywords “*autoimmune thyroiditis*” and “*Hashimoto thyroiditis*”. To ensure high confidence in disease relevance, genes were filtered using the GeneCards Gifts Score, which integrates genetic, expression, functional, and clinical evidence. Only genes with a Gifts ≥ 60 were included, resulting in a final list of 1047 AIT-related genes.

#### 4.8.2. Identification of Vitamin D Targets

Targets associated with vitamin D (Cholecalciferol D_3_: PubChem CID: 5280795) were collected from the SwissTargetPrediction platform (http://www.swisstargetprediction.ch (accessed 2 November 2025), a widely cited and validated ligand-based target prediction tool. This web server has been used in numerous pharmacological studies for target prediction and network construction, where the focus is on generating a high-confidence subset of targets for further exploratory analysis rather than an exhaustive list of all possible interactions [32,33]. To enhance confidence in downstream analyses, we applied a probability cutoff of >0.1 for predicted targets, focusing on high-confidence interactions. This approach yielded 19 vitamin D_3_-associated targets.

The obtained 19 targets were collected and standardized according to UniProt gene symbols. The overlap between vitamin D–related and AIT-associated genes was visualized using a Venn diagram generated via https://bioinformatics.psb.ugent.be/webtools/Venn/ (accessed 2 November 2025). The analysis demonstrated that 10 genes were common to both datasets, as presented in Figure 5.

#### 4.8.3. Protein–Protein Interaction (PPI) Network Construction

The overlapping target genes were uploaded to the STRING database (version 12.0; https://string-db.org/) (accessed 8 November 2025) with the species restricted to *Homo sapiens* and a medium interaction confidence score of 0.4. This threshold was selected to balance network coverage with interaction reliability in an exploratory setting. Interaction data were exported in SIF format and visualized using Cytoscape version 3.10.4 (https://cytoscape.org/, accessed on 8 November 2025).

Network topological parameters, including degree, betweenness centrality, and closeness centrality, were calculated to identify highly connected nodes. Hub gene prioritization was further performed using the cytoHubba plugin, applying the Degree method, and the top five ranked genes were selected as core targets [33]. Given the relatively small number of overlapping genes, hub identification was interpreted cautiously and considered hypothesis-generating rather than definitive.

#### 4.8.4. Functional Enrichment Analysis

Gene Ontology (GO) and Kyoto Encyclopedia of Genes and Genomes (KEGG) pathway enrichment analyses were performed using DAVID (https://davidbioinformatics.nih.gov/ retrieved on 11 November 2025) and SHINY GO 0.85.1 (http://bioinformatics.sdstate.edu/go/, accessed on 11 November 2025). Enrichment results included the biological processes, molecular functions, and signaling pathways associated with steroid hormone biosynthesis, estrogen and androgen signaling, lipid metabolism, immune regulation, cytokine response, and inflammation, which were prioritized for interpretation, given their relevance to thyroid autoimmunity and vitamin D.

Gene Ontology (GO) and Kyoto Encyclopedia of Genes and Genomes (KEGG) pathway enrichment analyses were conducted using DAVID (https://davidbioinformatics.nih.gov/, accessed on 11 November 2025) and SHINY GO v0.85.1 (http://bioinformatics.sdstate.edu/go/), accessed on 11 November 2025. Enriched biological processes, molecular functions, and signaling pathways were identified based on adjusted *p*-values.

Given the limited size of the overlapping gene set, enrichment analyses were interpreted as exploratory. Pathways related to steroid hormone biosynthesis, estrogen and androgen signaling, lipid metabolism, immune regulation, cytokine signaling, and inflammatory responses were prioritized for interpretation due to their established relevance to thyroid autoimmunity and vitamin D biology.

#### 4.8.5. Molecular Docking

Molecular docking was performed to assess the binding affinity of vitamin D_3_ (cholecalciferol) toward the top-ranked hub proteins identified in the network analysis, including: CYP19A1 (PDB: 3S79), CYP17A1 (PDB: 3RUK), and ESR1 (PDB: 1XP1). Docking simulations were carried out using the Glide module in Maestro (Schrödinger Release 2024-1). Protein structures were retrieved from the Protein Data Bank and prepared using the Protein Preparation Wizard, including hydrogen atom addition, optimization of protonation states at physiological pH, correction of missing side chains, and restrained energy minimization. For CYP19A1 and CYP17A1, the heme prosthetic group was removed prior to docking to enable assessment of direct ligand–protein interactions, consistent with prior in silico docking studies on cytochrome P450 enzymes [34].

Vitamin D_3_ and the co-crystallized ligands were prepared using LigPrep, generating multiple low-energy conformations. Docking validation was performed by redocking the native ligands into their respective binding sites, and pose accuracy was assessed by calculating the root-mean-square deviation (RMSD) between docked and crystallographic ligand conformations. Docking simulations were conducted using the Glide Extra Precision (XP) protocol. More details on the preparation methods for the protein and Vitamin D_3_ can be found in these studies [33,35].

Docking scores and interaction patterns, including hydrogen bonding, hydrophobic contacts, and π–π interactions, were analyzed. Binding poses were further visualized and analyzed using https://discover.3ds.com/discovery-studio-visualizer-download accessed on 20 November 2025). Docking results were interpreted as indicative of potential ligand–protein binding feasibility rather than direct evidence of biological activity.

#### 4.8.6. Molecular Dynamics Simulations (MDS)

Molecular dynamics (MD) simulations were performed to evaluate the structural stability and dynamic behavior of the vitamin D_3_–protein complexes identified by molecular docking. Simulations were carried out using GROMACS (version 2025.1) with the CHARMM36 all-atom force field applied to the proteins. Ligand parameters were generated using the SwissParam server (https://swissparam.ch/) accessed on 5 February 2026 [33]. Each protein-vitamin D_3_ complex was solvated in a cubic box using the TIP3P water model, and counterions (Na^+^/Cl^−^) were added to neutralize the system. Energy minimization was conducted using the steepest descent algorithm, followed by equilibration under constant volume (NVT) and constant pressure (NPT) conditions. Production MD simulations were then performed for 100 ns under periodic boundary conditions. Trajectory analyses included root-mean-square deviation (RMSD), root-mean-square fluctuation (RMSF), radius of gyration (Rg), solvent-accessible surface area (SASA), and hydrogen-bond occupancy, which were used to assess complex stability and conformational dynamics. Binding free energies were further estimated using the MM/GBSA approach, providing energetic support for the docking-derived interactions.

#### 4.8.7. Statistical Analysis

Data analysis and visualization were performed using Python version 3.12 (Python Software Foundation, https://www.python.org) (accessed 10 September 2025, incorporating standard scientific libraries such as NumPy, Pandas, SciPy, and Matplotlib 3.8.2.

## 5. Conclusions

This study identified an important inverse association between serum vitamin D status and vaccine-induced antibody responses in patients with autoimmune thyroiditis, suggesting that vitamin D deficiency may be associated with heightened humoral immune reactivity following COVID-19 vaccination. Vitamin D-deficient individuals exhibited higher anti-RBD IgG levels and greater variability in thyroid autoantibody profiles, consistent with a pattern of immune dysregulation in the context of thyroid autoimmunity.

Network pharmacology analysis indicated that vitamin D-related targets were enriched in pathways linked to estrogen signaling, steroid biosynthesis, prolactin signaling, and hormone-regulated processes, providing a plausible endocrine–immune framework relevant to autoimmune thyroid disease. Complementary molecular docking supported the structural compatibility of vitamin D_3_ with key endocrine-related proteins, including ESR1, CYP19A1, and CYP17A1, suggesting potential interactions with hormonal regulatory pathways. Molecular dynamics simulations further supported the stability of these interactions over time, reinforcing the biological plausibility of the proposed mechanisms.

Together, these clinical and computational results suggest that vitamin D status may influence post-vaccination immune responses in autoimmune thyroiditis through modulation of endocrine–immune crosstalk. While causality cannot be inferred from the present study, the results support further investigation into the role of vitamin D in immune regulation within autoimmune populations. Longitudinal and interventional studies will be required to validate these observations and to clarify the potential contribution of micronutrient status to immune balance and autoimmune stability.

## Figures and Tables

**Table 1:** Functional enrichment results.

Enrichment FDR	nGenes	Pathway Genes	Fold Enrichment	Pathway	Genes
0.023366	1	20	116.29	Path:hsa00100 Steroid biosynthesis	CYP27B1
0.024618	1	23	101.1217391	Path:hsa00900 Terpenoid backbone biosynthesis	HMGCR
0.966 × 10^−6^	3	71	98.27323944	Path:hsa04917 Prolactin signaling pathway	CYP17A1, ESR1, ESR2
0.002181	2	52	89.45384615	Path:hsa04913 Ovarian steroidogenesis	CYP17A1, CYP19A1
0.002403	2	63	73.83492063	Path:hsa00140 Steroid hormone biosynthesis	CYP17A1, CYP19A1
0.004455	2	96	48.45416667	Path:hsa01522 Endocrine resistance	ESR1, ESR2
0.006534	2	138	33.70724638	Path:hsa04915 Estrogen signaling pathway	ESR1, ESR2
0.001337	3	215	32.45302326	Path:hsa05207 Chemical carcinogenesis-receptor activation	ESR1, ESR2, AR
0.006564	2	148	31.42972973	Path:hsa05224 Breast cancer	ESR1, ESR2
0.011236	2	218	21.33761468	Path:hsa04081 Hormone signaling	ESR1, ESR2
0.006231	3	529	13.18979206	Path:hsa05200 Pathways in cancer	ESR1, ESR2, AR
0.010073	4	1556	5.978920308	Path:hsa01100 Metabolic pathways	CYP17A1, CYP19A1, CYP27B1, HMGCR

**Table 2:** XP Docking scores and RMSD values of vitamin D_3_ and native ligands docked into CYP19A1, CYP17A1, and ESR1.

Proteins	Ligands/Vitamin D3	XP Score (Kcal/Mol)	RMSD (Å)
CYP19A1 PDB:3S79	Androstenedione	−8.234	/
	vitamin D3	−7.382	2.146
CYP17A1 PDB:3RUK	Abiraterone	−8.752	/
	vitamin D3	−7.289	1.953
ESR1 PDB:1XP1	Ligand (AIH)	−7.406	/
	vitamin D3	−8.016	0.007

**Table 3:** Predicted molecular interactions of vitamin D_3_ with CYP19A1, CYP17A1, and ESR1.

Compound	Protein	H-Bond	Number	Distance	Hydrophobic	Number	Distance
Vitamin D3	CYP19A1 PDB:3S79	LEU372MET374	2	[2.01–2.52]	ILE133 TRP224 MET364 VAL370 PHE430 CYS437 LEU477	8	[4.45–5.40]
	CYP17A1 PDB:3RUK	/	/	/	ALA113 ILE206 ALA302 VAL366 ALA367 ILE371 PHE435 CYS442 VAL482	10	[3.49–5.43]
	ESR1 PDB:1XP1	LYS531	2	[1.99–2.89]	ALA350 LEU525 CYS530 LEU536 MET343 LEU346 LEU349 TRP383 PHE404	15	[3.31–5.44]

**Table 4:** Average values of root mean square deviation (RMSD), Root mean square fluctuation (RMSF), Radius of gyration (Rg, and Solvent-accessible surface area (SASA).

Metric	CYP19A1-Vitamin D_3_	CYP17A1-Vitamin D_3_	ESR1-Vitamin D_3_
RMSD (nm)	0.130	0.135	0.240
RMSF (nm)	0.126	0.114	0.157
Rg (nm)	2.315	2.337	1.885
SASA (nm^2^)	222.974	230.901	140.650

**Table 5:** Molecular Mechanics/Generalized Born Surface Area (MMGBSA) Binding Energy Components.

Energy Component	CYP17A1-Vitamin D3	CYP19A1-Vitamin D3	ESR1-Vitamin D3
VDWAALS	−47.27	−3.84	−26.23
EEL	−13.73	−0.5	−2.89
EGB	26.96	2.1	10.12
ESURF	−6.36	−0.51	−3.16
GGAS	−61	−4.34	−29.12
GSOLV	20.6	1.59	6.97
Total ΔG	−40.4	−2.75	−22.15

## Data Availability

Due to ethical and privacy restrictions, the clinical data are not publicly available. Data may be provided by the corresponding author upon reasonable request.
